# The Utility of Novel Renal Biomarkers in Assessment of Chronic Kidney Disease of Unknown Etiology (CKDu): A Review

**DOI:** 10.3390/ijerph17249522

**Published:** 2020-12-18

**Authors:** T.D.K.S.C. Gunasekara, P. Mangala C.S. De Silva, Chula Herath, Sisira Siribaddana, Nipuna Siribaddana, Channa Jayasumana, Sudheera Jayasinghe, Maria Cardenas-Gonzalez, Nishad Jayasundara

**Affiliations:** 1 Department of Zoology, Faculty of Science, University of Ruhuna, Matara 81000, Sri Lanka; gunasekara.sc166@fgs.ruh.ac.lk; 2 Renal Division, Brigham and Women’s Hospital, Harvard Medical School, Boston, MA 02115, USA; mariana.cardenas@conacyt.mx; 3 Department of Nephrology, Sri Jayawardenapura General Hospital, Sri Jayawardenepura Kotte 10100, Sri Lanka; chulaherath@gmail.com; 4 Department of Medicine, Faculty of Medicine and Allied Sciences, Rajarata University of Sri Lanka, Mihintale 50300, Sri Lanka; sisira.siribaddana@gmail.com; 5 Department of Parasitology, Faculty of Medicine and Allied Sciences, Rajarata University of Sri Lanka, Mihintale 50300, Sri Lanka; nipunas@gmail.com; 6 Department of Pharmacology, Faculty of Medicine and Allied Sciences, Rajarata University of Sri Lanka, Mihintale 50300, Sri Lanka; channajayasumana@gmail.com; 7 Department of Pharmacology, Faculty of Medicine, University of Ruhuna, Matara 81000, Sri Lanka; sudheerasj@med.ruh.ac.lk; 8 Mexican Council of Science and Technology, Consejo Nacional de Ciencia y Tecnología, Mexico City 03940, Mexico; 9 The Nicholas School of the Environment, Duke University, Durham, NC 27708, USA; nishad.jayasundara@duke.edu; 10 The School of Marine Sciences, University of Maine, Orono, ME 04469, USA

**Keywords:** CKD, CKDu, novel biomarkers, renal function, rural farmers, Sri Lanka

## Abstract

Chronic Kidney Disease (CKD) is a globally prevalent non-communicable disease with significant mortality and morbidity. It is typically associated with diabetes and hypertension; however, over the last two decades, an emergence of CKD of unknown etiology (CKDu) has claimed thousands of lives in several tropical agricultural communities. CKDu is associated with gradual loss of renal function without initial symptoms until reaching complete kidney failure and eventually death. The most impacted are young adult males of lower socio-economic strata. Since the disease progression can be successfully attenuated through early detection, the development of superior screening and management measures is of utmost importance. In contrast to the conventional biomarkers, novel biomarkers with improved sensitivity and specificity are being discussed as promising tools for early diagnosis of the disease. This review summarizes emerging novel biomarkers used in assessing CKD and discusses the current utility and diagnostic potential of such biomarkers for CKDu screening in clinical settings of different communities impacted by CKDu. Our goal is to provide a framework for practitioners in CKDu impacted regions to consider the use of these novel biomarkers through this synthesis. The increased use of these biomarkers will not only help to validate their diagnostic power further and establish potential prognostic value but may also provide critical insights into sites and mechanisms of renal damage.

## 1. Background

Chronic Kidney Disease (CKD) is a non-communicable disease that carries a high morbidity and mortality and affects more than~10% of the global population. CKD is defined as “abnormalities of kidney structure or function, present for more than three months” [1]. Clinically, reduction of Glomerular Filtration Rate (GFR) to less than 60 mL/min/1.73 m^2^ or increase in albumin to creatinine ratio (ACR ≥ 30 mg/g (3 mg/mmol)) is the main criteria for the diagnosis. The leading etiologies of the disease worldwide are type 2 diabetes mellitus, hypertension and chronic glomerulonephritis [2,3]. The estimated number of disability-adjusted life years (DALYS), a measurement of health and wellness lost, attributable to CKD has increased by 26% globally from 1990 to 2013 [4].

**Figure 1:**
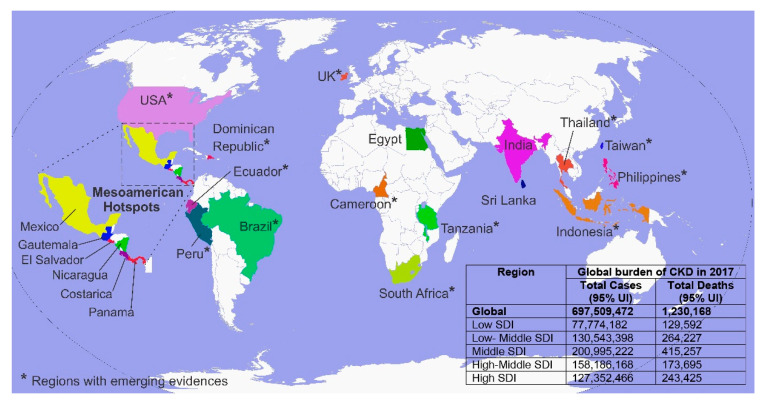
Endemic chronic kidney disease of unknown etiology (CKDu) hotspots and regions with emerging evidence of CKDu and chronic kidney disease (CKD) of nontraditional causes (CKDnT) around the world. Global burden is illustrated as all forms of CKD, including CKDu in countries of low to high socio-demographic index (SDI)-based economic strata.

In contrast to CKD associated with typical risk factors, a new form of renal disease has emerged in several parts of the world, including in tropical farming communities in Central American and South Asian countries [5] (Figure 1). Both CKD and chronic kidney disease of unknown etiology (CKDu) are characterized by a progressive decline in renal function. However, the exact causes of this disease are highly debated, and given this ambiguity, it is commonly referred to as chronic kidney disease of unknown etiology (CKDu) or, in some instances, as chronic interstitial nephritis in agricultural communities (CINAC) [6]. Among other factors, epidemiological studies suggest a role for environmental contaminant exposures, heat stress, and dehydration associated with farming, infections, and mycotoxins. Recent studies posit that synergistic effects of multiple environmental factors coupled with genetic predisposing may contribute to the initiation, continuous renal injury, and kidney dysfunction progression.

**Figure 2:**
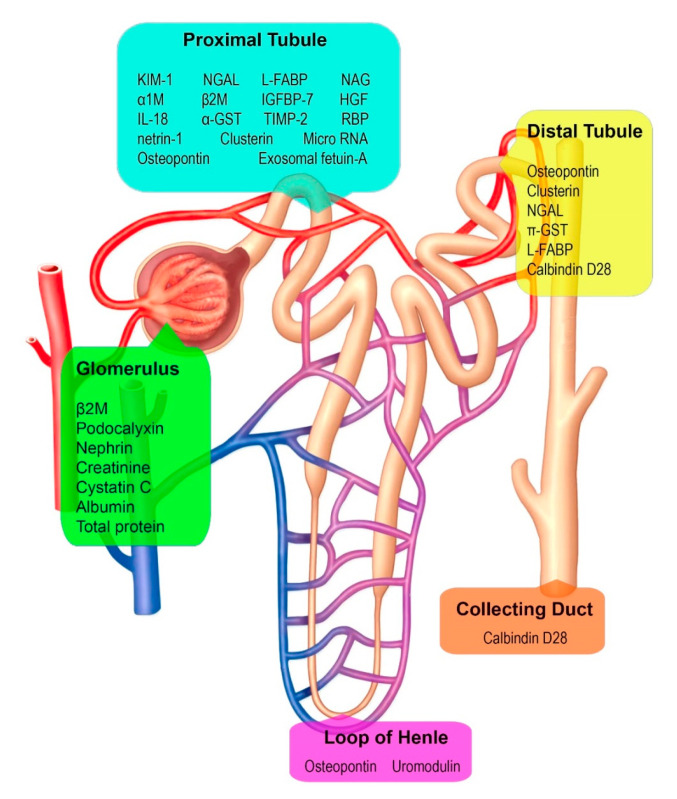
Macromolecules that may serve as biomarkers and their sites of expression. (KIM-1: Kidney Injury Molecule-1; NGAL: Neutrophil Gelatinase-Associated Lipocalin; TIMP-2: Tissue Inhibitor Metalloproteinase-2; IGFBP-7: Insulin- like Growth Factor Binding Protein-7; Pi-GST: Glutathione S-Transferase; NAG: N-acetyl-beta-D-glucosaminidase; IL-18: Interleukin-18; RBP: Retinol Binding Protein; L-FABP: Liver type Fatty Acid Binding Protein; α1M: α1- Microglobulin; β2M: β2- Microglobulin; HGF: Hepatocyte Growth Factor.).

A major challenge in mitigating CKDu is the lack of early diagnostic assays, and the potential to use renal injury markers as early indicators of CKDu manifestation is beginning to be explored. In CKDu patients, the main clinical manifestations such as elevated serum creatinine, protein excretion and urinary albumin to creatinine ratio (ACR), become apparent only after a significant damage to the renal tissues. However, renal tissues express a variety of molecules such as KIM-1, NGAL, and NAG in response to renal injury (Figure 2). These may have the potential to reflect renal damage earlier than conventional markers with a high degree of specificity. They are indeed emerging as potential early predictors of renal damage in CKD and may contribute to the early diagnosis of CKDu. However, convenient assessment strategies and precise reference standards have not been established yet; hence the transformation of these novel biomarkers into clinical screening tools in CKDu requires further studies, and their prognostic power remains to be validated. The objective of this review is to highlight the current use and limitations of novel biomarkers in CKDu studies to provide an initial framework for practitioners in CKDu affected communities when considering the use of these novel biomarkers.

## 2. Chronic Kidney Disease of Unknown Etiology (CKDu)

Similar to most other forms of CKD, CKDu is also asymptomatic until later stages. It is initially relatively non-proteinuric (less than 1 g/24 h), and early identification has been challenging. Histopathology of patients from later stages shows a chronic tubulo-interstitial disease with secondary glomerulosclerosis [7].

CKDu incidences first emerged in several Mesoamerican countries, including El Salvador, Nicaragua, Guatemala, Mexico, Panama, and Costa Rica [8], Andhra Pradesh in India, the El-Minia Governorate in Egypt, and several provinces of Sri Lanka [9]. Current evidence presented in various studies suggests incidences in several other South East Asian countries, including Thailand [10], Indonesia [11], the Philippines, and Taiwan; in Tanzania, South Africa, Cameroon, Brazil, Peru, Ecuador, and Dominican Republic [12]; in parts of the United States of America [11] and Belfast in the United Kingdom [13]. Clinical, histological, biochemical, environmental, and socio-economic factors associated with CKDu are comparable across its geographical distribution and are considered a rapidly growing public health concern worldwide [14,15]. CKDu is predominant in rural agricultural communities, disproportionately affecting young to middle-aged male farmers [16,17].

The disease is characterized by prominent histopathological features that tend to be consistent over the global CKDu hotspots. In a study with Indian CKDu patients, glomerular sclerosis, interstitial infiltration with lymphocytic infiltration, interstitial fibrosis, and tubular atrophy were noted prominently. Importantly, significant histological changes have been noted in early-stage CKDu patients [18]. In a study with 64 renal biopsies from CKDu patients in Sri Lanka, interstitial fibrosis and tubular atrophy with or without nonspecific interstitial mononuclear cell infiltration were the dominant histopathological manifestations. In addition, glomerular sclerosis, glomerular collapse, and features of vascular pathology such as fibrous intimal thickening and arteriolar hyalinosis were also noted commonly [19]. The same histopathological and biochemical profiles have been observed in Mesoamerica [15,20].

Since its emergence, CKDu has claimed thousands of lives in Central America and South Asia [21,22], devastating impacted rural communities. Most affected communities are predominantly rural and rarely undergo regular medical checkups due to socio-economic limitations as well as the absence of other risk factors (e.g., type 2 diabetes and hypertension). The disease predominantly affects males, and lack of optimal disease management strategies has led to end-stage renal failure and death in most cases [23]. As illustrated in Figure 1, CKDu prevalence has increased across the globe and several CKDu hotspots have emerged, making it a global public health concern [24].

CKDu hotspots are defined based on the high prevalence of CKD attributed to unexplained causes. However, characterizing precise CKDu prevalence has been difficult due to several factors, including the ambiguity in defining geographical boundaries of impacted and non-impacted regions. In most community studies, declined renal function in the absence of diabetes mellitus, hypertension, and glomerular diseases is treated as CKDu or CKD of nontraditional causes. These clinical manifestations associated with CKDu were confirmed in a recent study with CKDu patients in Sri Lanka [25]. Nonetheless, several community studies conducted in 2019 in CKDu affected regions indicate the gravity of this epidemic. In one such study conducted in the disease endemic North Central Province in Sri Lanka, CKDu prevalence among adults was reported as 6.0% [17], while it was estimated as 13.3% among adults in the Uddanam region in India [26]. Similar cross-sectional studies of CKDu in other farming communities such as among the west Javanese rice farmers in Indonesia (18.6%) [11], residents of León in Nicaragua (7.4–8.4%) [27], suggest a high prevalence of this disease, although these statistics vary depending on the study. Another key limitation is the lack of a standardized disease diagnosis approach as well as the lack of intensive data collection from population surveys. An important step towards facilitating better health policies and intervention strategies is developing early detection markers of CKDu.

## 3. Etiology of CKDu

Although the exact etiology is yet to be determined, several environmental, socio-economic, and occupational factors are strongly associated with CKDu. Studies have suggested a role for a multitude of contributing factors such as chronic low dose exposure to multiple heavy metals and agrochemicals [28,29], heat stress and recurrent dehydration [30,31], nephrotoxic pharmaceuticals [32], high fluoride levels and water hardness [33], hyperuricemia and hyperuricosuria [34], leptospirosis [35], and genetic predisposition [36]. Heat stress, coupled with dehydration, are considered key driving factors in Mesoamerican CKDu patients [12,37]. However, a recent meta-analysis suggested that pesticide exposure, heat stress, and pharmaceutical use were not associated with CKDu in Mero-America [8]. In South Asia, environmental contaminants are believed to play a significant role in disease initiation and progression [29,38]. Notably, a recent review of studies focused on the etiologies of CKDu has shown that many exposures are heterogeneous and vary by region [39]. Accordingly, a combination of factors are likely contributors to this disease at different stages [9], and below, we summarize the three most prominently discussed drivers of CKDu.

### 3.1. Heat Stress and CKDu

According to the heat stress/dehydration hypothesis, recurrent mild acute kidney injury caused by recurrent work-associated dehydration episodes leads to CKD [40]. Dehydration causes hyperosmolarity, which activates the aldose reductase enzyme in the proximal tubule of the nephron. This enzyme is involved in the polyol pathway of glucose metabolism that converts glucose to fructose. Metabolism of fructose by fructokinase results in oxidative stress leading to tubular injury. In addition, this hyperosmolarity increases vasopressin, affecting kidney tubules. The predominant distribution of global CKDu hotspots in warmer climates favors this hypothesis. Indeed, CKDu is commonly observed in individuals working strenuously in agricultural fields under extreme heat and are dehydrated [12]. While some studies have argued that there is no clear association between ambient temperature and CKDu [41], this theory’s more detailed rebuttal has been published recently [42]. Thus, the hypothesis warrants further validation in different communities through in-depth studies.

### 3.2. Environmental Contaminants, Water Quality, and CKDu

Several classes of naturally occurring and anthropogenic chemical compounds have been discussed to play a significant role in CKDu. This hypothesis was further supported by a recent study that examined 34 renal biopsies of patients with CKD stage 2–3 (who were diagnosed as CKDu patients) from Sri Lanka, India, El Salvador, and France. Lysosomal proximal tubulopathy with abnormal enlarged dysmorphic lysosomes containing dispersed dark electron-dense aggregates, under light and electron microscopy, indicating a toxin-induced proximal tubular nephropathy in CKDu patients [6,43]. Indeed, varying levels of heavy metals such as mercury, cadmium and lead, arsenic, vanadium, and other agrochemical constituents as well as other nephrotoxic compounds have been found in soil, drinking water, and food in CKDu endemic regions [29,44,45]. In contrast, in a recent study in CKDu prevalent areas in Sri Lanka, analysis of urine, hair, and renal tissue samples did not provide evidence to support cadmium or arsenic toxicity in CKDu patients. Further, Cd and as levels in water and rice samples were within the recommended levels [46]. Animal model studies demonstrated that chemical mixtures found in the environment might induce renal damage and alter kidney development [29]. Additionally, the same substances have been found in urine, blood, hair, and nail samples collected from affected populations [14,47,48], providing strong evidence for an association with environmental toxins with renal diseases, including CKDu. Further, studies from multiple geographic regions suggest clear associations of groundwater fluoride and water hardness with the onset of renal diseases, including interstitial tubule damage [49] coupled with experimental validation through animal studies [33]. Certain nephrotoxic mycotoxins (e.g., aflatoxins, ochratoxins, fumonisins) and phytotoxins such as aristolochic acid, are other environmental factors known to cause chronic tubule-interstitial disease. Among the nephrotoxic agents, a role for aristolochic acid is well documented. Aristolochic acid nephropathy (AAN), formerly known as “Chinese herbal nephropathy” has accounted for a number of cases of chronic renal failure in countries where traditional herbal medicines are widely used [50,51]. Balkan endemic nephropathy (BEN), a severe kidney disease reported in the Balkan Peninsula, is also attributed to AAN. Histology studies show that AAN results in an interstitial fibrosis with a typical corticomedullary gradient and tubular atrophy [52], which has been validated in several animal studies [53,54]. However, there is a lack of clear evidence linking them to CKDu in South Asia and Mesoamerica. Further research is warranted to examine mechanisms of toxicity, underlying initiation and propagation of renal damage by toxic substances.

### 3.3. Infectious Origin

Two studies have shown a possible link between CKDu and an infectious etiology. One study demonstrated elevated levels of Hantavirus IgG antibodies in CKDu patients in Sri Lanka [55], and the other indicated leptospirosis as a risk factor for CKDu in several farming communities [35]. Although these data may provide an association between CKDu and an infectious etiology, further research is required to substantiate a causal link.

## 4. Renal Biomarkers

The National Institutes of Health (NIH), USA defines a biomarker as “a characteristic that is objectively measured and evaluated as an indicator of normal biological processes, pathogenic processes, or pharmacologic responses to a therapeutic intervention” [56]. In a clinical context, biomarkers can be applied as tools for screening, diagnosing, and monitoring diseases as well as assessing the response of therapeutic interventions [57].

The progression of renal diseases can be successfully alleviated if diagnosed at the potentially treatable and reversible earlier phases. Thus, early detection is of utmost importance, especially for CKDu patients. Biomarkers play a distinct role in aiding the prediction of disease progression and monitoring therapeutic outcomes, and developing more potent biomarkers has become essential in kidney disease management strategies [58].

Cellular stress responses in various renal structures produce several macromolecules at different locations that can serve as markers of kidney dysfunction or damage (Figure 2). For example, a wide spectrum of molecules including urinary and blood-borne proteins and recently discovered micro RNAs might serve as biomarkers in kidney disease screening. However, the sensitivity, specificity, and predictive value of these biomarkers are diverse.

At present, the CKDu epidemic in predominantly agricultural communities has been characterized using conventional renal biomarkers such as serum creatinine (SCr), cystatin C, blood urea nitrogen (BUN), estimated GFR (eGFR), and albumin to creatinine ratio (ACR). These markers have deficiencies in sensitivity and/or specificity to detect kidney injury (or response to injury), especially prior to significant renal function loss. For example, CKDu is a relatively non-proteinuric condition and will often not show a significant elevation of ACR. Furthermore, the elevation of SCr occurs only when significant renal damage has occurred. Hence, the utility of more specific and sensitive biomarkers and better-designed population studies are important, especially for an early diagnosis.

In contrast to conventional renal damage markers, emerging novel biomarkers can serve as early predictors of renal diseases and help locate the site of injury [59,60]. Despite the emerging use of a wide range of novel biomarkers in acute kidney injury and CKD, such approaches are just beginning to be applied in monitoring CKDu. Kidney injury molecule -1 (KIM-1), neutrophil gelatinase-associated lipocalin (NGAL), α1-microglobulin (A1M), β2- macroglobulin (B2M), N-acetyl-beta-D-glucosaminidase (NAG), clusterin (CLU), osteopontin (OPN), and cystatin-C (CysC) are promising examples of new biomarkers. In 2018, the Food and Drug Administration (FDA) approved the qualification of KIM-1, NGAL, NAG, CLU, CysC, and OPN as biomarkers to facilitate the detection of renal tubular injury in phase 1 trials in healthy individuals [61]. A summary of these novel kidney injury biomarkers’ characteristics and clinical significance and their utilization in CKDu contexts across the world are given in Table 1 and the site of release is illustrated in Figure 2. These can potentially help to (i) predict and monitor CKDu outcomes in preclinical and clinical settings and (ii) gain critical insights into renal damage sites at the early stages of the disease.

## 5. Current Use of Novel Biomarkers in CKDu Hotspots and Their Diagnostic Potential

### 5.1. Mesoamerica

**Figure 3:**
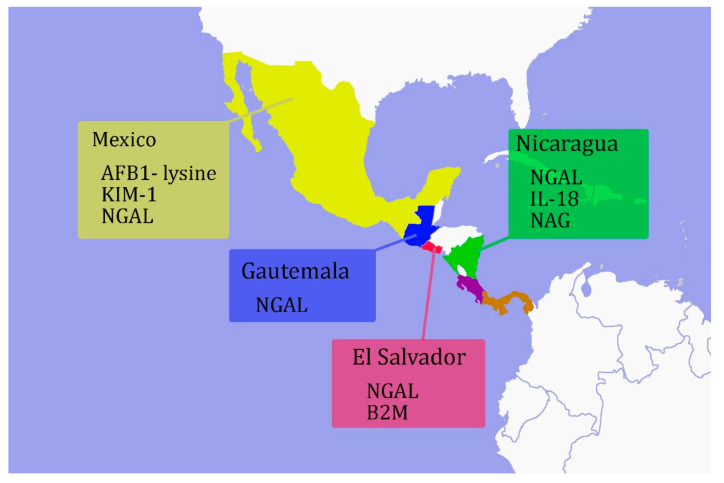
Overview of commonly used renal biomarkers for screening CKDu affected communities in the Mesoamerican region.

Mesoamerican countries with CKDu include Mexico, Belize, Guatemala, El Salvador, Honduras, Nicaragua, Peru, and the northern part of Costa Rica. The first published study on Mesoamerican CKDu appeared in 2002, describing 205 newly dialyzed patients from 1999–2000 in El Salvador. As it is commonly observed today, the patients were predominantly men (87%), working in the sugarcane industry (63%), with an average age of 51 years. A majority of them (73%) had been exposed to agrochemicals. The cause of kidney failure was unknown, and it was proposed to be of toxic origin [62]. Subsequently, similar patients were recorded from several Mesoamerican countries. Among diverse Mesoamerican farming communities, impaired renal function has been characterized in terms of conventional renal biomarkers, including eGFR, SCr (serum creatinine), and ACR [63,64]. However, novel biomarkers are beginning to be used in Mesoamerica, and here we describe some of these studies, emphasizing their use and diagnostic potential (Figure 3).

A recent study in a CKDu impacted community established renal dysfunction based on eGFR and urinary NGAL [65]. The population composed of 350 apparently healthy young adults in the age range 18–30 from affected areas in northwest Nicaragua, of whom 74% of participants were males working outdoors. Serum and urine creatinine, urinary albumin, urinary NGAL (uNGAL), serum cystatin C, and eGFR were monitored as diagnostic measures during the follow-up of two years. Based on the baseline eGFR values, participants were divided into three groups, group 1 (established renal dysfunction), group 2 (rapidly declining renal function), and group 3 (control). Group 1 individuals showed higher baseline uNGAL levels in comparison to other groups. Furthermore, 16% of group 1 and 4.7% of group 3 participants showed urinary ACR ≥ 30 mg/g. A model based only on a single eGFR measurement was predictive of renal dysfunction in group 1 compared to group 3, and the addition of uNGAL showed no improvement in predictive value. While looking for an effective model for predicting the decline in renal function, using eGFR at baseline, 6 months, and 12 months showed the best outcomes, and incorporation of uNGAL in the model showed the same predictive capacity. The findings indicate that a minimum follow-up period of one year with measurements starting from baseline and then at six-month intervals is necessary to differentiate between the progressive loss of eGFR and stable kidney function [66]. However, this study does not provide strong evidence for any added value of uNGAL in CKDu screening.

Similar findings were evident in a cross-sectional study with 189 sugar cane cutters (18–49 years old) in El Salvador. Elevated SCr and uric acid levels were commonly observed in both males and females. Reduced eGFR was observed in slightly older individuals. Elevated BUN levels, SCr, and uNGAL were observed in participants with proteinuria compared to the non-proteinuric. Serum creatinine, urea nitrogen, and uric acid showed significant cross-shift (during the work shift) increases. The participants’ working hours were in the range 1.4 to 11 h and the day temperature ranged between 34 to 42 °C. Thus, recurrent dehydration from strenuous work under high heat exposure might be a factor for observed cross shift renal stress [30]. However, uNGAL decreased over their work shift. Similarly, Sorensen et al. (2019) [67] reported a significant decrease in uNGAL across Guatemalan sugarcane workers’ work shift. This decrease was coupled to a marked decline in eGFR across the work shift and was significantly associated with BUN, increased uric acid, increase in temperature, and serum osmolality. Importantly, this increase in uNGAL across the work shift was significantly correlated with a decline in eGFR, indicating the utility of uNGAL as a marker of declining renal function. The same association between uNGAL and declining eGFR was observed in several community-based cohort studies in Mesoamerican regions with CKDu risk [68,69].

These findings were further elaborated in another longitudinal study with 29 male sugarcane cutters aged 17–38. Compared to the baseline records before the beginning of harvesting season, the levels of SCr, BUN and urinary NGAL were increased at the end of the harvesting season of nine weeks. Particularly remarkable was the decrease of eGFR observed during this period, indicating a considerable renal function impairment during the harvesting season [37].

In Northwestern Nicaragua, blood and urine samples were analyzed in 284 sugarcane workers at the beginning of the harvesting season as well as six months later. The group’s mean eGFR was 113 mL/min/1.73 m^2^ at the baseline and albuminuria was observed in some participants. In comparison to non-field workers, field workers reported elevated levels of NGAL and IL-18 during the harvesting season. Also, workers with the highest elevations in NGAL and NAG levels showed a decline in eGFR during harvesting season. Although no overall effect of hydration was significant, electrolyte solution consumption was associated with lower mean NGAL and NAG levels [69]. The study proposed that exposure to heat with recurrent dehydration is a potential causative factor, although a role for nephrotoxins was not ruled out. Notably, the study provides evidence to support elevated levels of NGAL, NAG, and IL-18 as diagnostic biomarkers of CKDu.

Novel and conventional biomarkers were used in another small descriptive study with 10 females diagnosed as CKDu stage 2 and 3 in El Salvador’s agricultural communities. Their kidney biopsies showed renal damage restricted mostly to the tubulo-interstitium, and increased urinary excretion of B2M and NGAL was observed [70].

To investigate kidney disease initiation early in childhood, kidney injury biomarkers were assessed in 200 young school students aged 12–18 from different CKDu affected regions of Nicaragua. Comparisons were made with some schools located in CKDu risk areas. The presence of urinary glucose and protein, hemoglobinuria, and leucocyte esterase were rarely observed, while dysuria was common. A higher incidence of increased urine concentration was observed in girls compared to boys. Generally, NGAL, NAG, and IL-18 levels were significantly higher in boys than girls. However, higher NGAL levels were observed in leukocyte esterase positive girls and mean IL-18 levels were also higher in girls with hematuria [71]. Another recent study with 210 primary and secondary school students (7–17 years of age) from regions with a high prevalence of CKDu along the pacific coast of Nicaragua, reported consistent findings. Urinary KIM-1, NGAL, and IL-18 levels of the students were higher than those reported from healthy children in the USA and Poland. Although urinary monocyte chemoattractant protein 1 (MCP-1), and chitinase-3-like protein 1 (YKL-40) were assessed, researchers could not establish a clear association with renal function. However, girls reported significantly higher levels of these five biomarkers in urine than the boys [72], which notably contrasts the male predominance among CKDU patient cohorts [71,72]. However, this sex effect on renal injury and related biomarker expression, has not been adequately studied. Particularly elevated NGAL, NAG, KIM-1 and IL-18 levels in urine were observed in the students from regions with higher CKDu and adult mortality rates. This indicates the potential early onset of kidney disease in CKDu endemic areas and the potential to use these markers as early diagnostic tools. A similar risk of impaired renal function was observed in a study with 1058 boys and 1057 girls (age < 18 years) from CKDu regions in El Salvador using conventional markers [73]. These findings are also consistent with data on Sri Lankan children in CKDu endemic areas, where they are at a higher risk of kidney impairment [74]. These data strongly support a role for nephrotoxins potentially initiating the disease early on and further illustrate the critical need for markers predictive of early-stage renal damage and/or dysfunction.

While few studies have examined links between toxic insults and CKDu in Mesoamerica, novel biomarkers have rarely been used in this context. However, a recent pilot study in indigenous Mexican women measured their exposure to aflatoxin-B1 (AFB1) and was assessed using AFB1-lysine as a biomarker. AFB1-Lys was found in the majority of the study population, indicating a serious toxic exposure. There was also a strong significant correlation of AFB1-lysine with KIM-1 and Cystatin C expression, but not with NGAL [75].

In Mesoamerica, leptospirosis was also proposed as a possible causative factor underlying CKDu. In a study involving 282 sugarcane workers, 47 endemic controls, and 160 workers from other industries, Leptospira sero-positive individuals were higher among sugarcane cutters than other groups. These sero-positive individuals had higher NGAL expression than the sero-negative candidates, suggesting an association between leptospirosis and Mesoamerican nephropathy [76].

In summary, the use of novel biomarkers in Mesoamerica mainly focused on agricultural workers and other established conventional renal markers. Urinary NGAL was the most frequently used novel biomarker combined with other markers such as NAG and AFB1-lysine. While these biomarkers, with a few exceptions, generally followed tends demonstrated by conventional markers indicating their diagnostic potential, further studies are needed to confirm their prognostic value. Future studies may consider including one or more of the biomarkers discussed in this review to establish their use as early markers of CKDu risk potentially.

### 5.2. Sri Lanka

**Figure 4:**
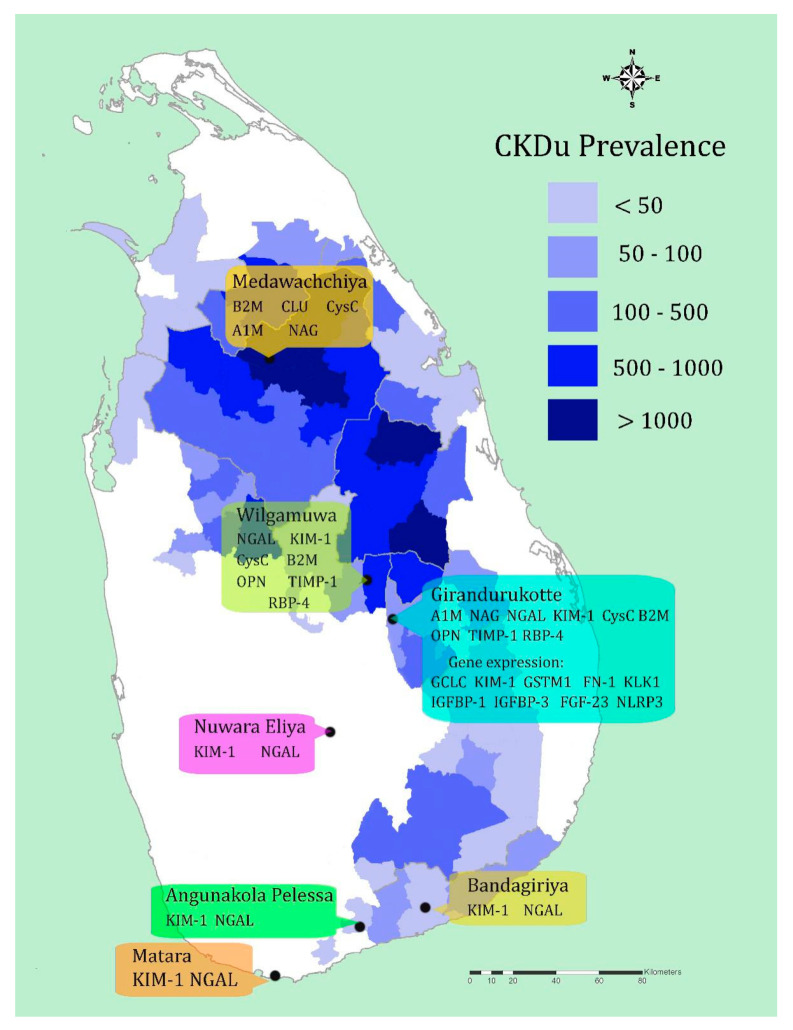
Regional distribution of CKDu in Sri Lanka and currently assessed biomarkers in different communities. Prevalence is illustrated in terms of the number of reported cases in the level of Divisional Secretariat Divisions.

CKDu in Sri Lanka was first identified in the early 1990s among the farming communities in the Anuradhapura district. The disease was predominantly observed among the middle-aged male farmers [77]. Since its recognition, the disease has emerged as a significant threat to inhabitants in the agricultural communities in 11 districts [78] (Figure 4) in the country’s dry zone. The disease is primarily characterized by conventional markers such as ACR, eGFR, and proteinuria [74,79], although recent studies have utilized novel biomarkers. Rathnayake et al. (2017) found Cystatin C as the best functional marker over creatinine and ACR in detecting CKDu in endemic regions in their attempt to select the best marker for community screening. However, the comparatively high analytical cost associated with Cystatin C (Supplementary Materials Table S1) tends to hinder its application in community screening [79]. Thus, identifying cost-effective novel biomarkers will be more economical for community screening, particularly with increasing disease prevalence.

The potential applicability of novel biomarkers KIM-1 and NGAL in CKDu characterization in Sri Lankan farming communities was first illustrated by De Silva et al. (2016) [80]. This study showed that NGAL and KIM-1 expression in urine is significantly correlated with high urinary ACR levels in the participants from CKDu endemic and emerging locations in comparison to healthy controls. Importantly, NGAL and KIM-1 levels were elevated in apparently healthy farmers with normal urinary ACR, indicating the potential early prognostic capacity of these markers in the CKDu context [77]. Elevated KIM-1 and NGAL were also found in CKDu non-endemic locations indicating early renal damage among rural farming communities.

Chung et al. (2017) [81] adopted a novel technique called micro-urine nanoparticle detection (μUNPD) and assessed urine samples of 42 candidates, including three acute kidney injury

(AKI) patients, 18 CKD patients from Western Province, and healthy participants from North Central Province in Sri Lanka. AKI patients showed an increase in urinary KIM-1 levels, and both KIM-1 and cystatin were elevated in CKD patients, suggesting that these biomarkers can be used for disease diagnosis.

In another study, a group of patients with CKD and CKDu from Girandurukotte (a CKDu endemic area) and healthy controls were examined for genetic biomarker-based differentiation of CKDu and CKD. Significant differences in expression of genes coding for insulin-like growth factor binding protein 1 (IGFBP1), KIM1, Glutathione-S-transferase mu 1 (GSTM1), glutamate-cysteine ligase catalytic subunit (GCLC) was observed in CKD and CKDu patients in varying degrees compared to healthy controls. In addition, significant correlations of differentially expressed genes with serum creatinine and, in turn, with eGFR were observed. The overall analysis suggested that a combined panel of IGFBP1, KIM1, GCLC, and GSTM1 genes could be used for early screening of CKDu and these genes in addition to fibronectin 1 (FN1), IGFBP3, and kallikrein 1 (KLK1) could be used for monitoring CKDu progression. However, further validation may be required prior to clinical use [82]. A similar study was conducted to assess gene expression changes associated with oxidative stress in CKD and CKDu patients in comparison to healthy controls from CKDu endemic and non-endemic areas. Compared to the controls from non-endemic areas, genes coding for glutamate-cysteine ligase catalytic subunit (GCLC), glutathioneS-transferasemu1 (GSTM1), fibroblast growth factor-23 (FGF23), and NLR family pyrin domain containing 3 (NLRP3) were upregulated in CKD and CKDu patients in varying degrees [83]. These studies indicate that redox imbalance may play a mechanistic role in initiation or progression of this disease, and oxidative stress markers may serve as early biomarkers of renal cellular dysfunction. However, oxidative stress markers are associated with many disease etiologies and may need significant validation prior to clinical use.

Several renal biomarkers’ predictive performance was assessed in a recent cross-sectional study incorporating CKDu patients and CKD patients along with healthy controls from endemic and non-endemic areas. Compared to healthy controls, CKDu and CKD patients showed elevated median levels of several biomarkers including α1-microglobulin, β2-microglobulin, cystatin C, and retinol binding protein 4 (RBP4) and NGAL in varying degrees. Further, in terms of specificity and sensitivity, A1M, KIM1, and RBP4 were found to be the best minimum marker combination for differentiation of CKD and CKDu from healthy controls. OPN, KIM1, and RBP4 combination showed a high diagnostic value for CKDu and CKD patients [84].

A comparative cross-sectional study with 37 CKDu patients and control groups from CKDu endemic and non-endemic areas also showed significantly elevated levels of several urinary biomarkers, including fibrinogen, CLU, CysC, and B2M in CKDu patients. Fibrinogen and B2M were the most significant markers in distinguishing CKDu patients from healthy controls. Urinary fibrinogen and B2M also showed positive correlations with arsenic and urinary mercury levels, respectively. In workers with lead exposure, urinary KIM1 showed a better correlation with lead levels in blood [85].

Urinary levels of B2M were assessed in a study with 30 CKDu patients and 30 controls, who were matched for age and sex from Medawachchiya, a CKDu endemic area in Sri Lanka. Urinary B2M was significantly higher in CKDu patients, and the mean urinary B2M level of CKDu patients showed a gradual increase with the progression of the disease as indicated by the deterioration of renal function. The study aimed to evaluate the effect of staple foods such as rice on kidney dysfunction as defined by urinary B2M levels, but no link was detected [86]. Levels of urinary biomarkers and cadmium exposure were investigated with a group of 106 CKDu affected patients in North Central Province. A control group of non-affected blood relatives of the selected CKDu patients and non-affected individuals from Japan served as controls. When compared to controls, urinary A1M was observed at a significantly higher level in CKDu stage 1 and a steady increase was observed through CKDu stage 1 to 3 (defined as eGFR stratification). Urinary NAG levels showed a significant difference only in CKDu stage 5 patients, suggesting A1M may be a useful early stage renal disease marker. In comparison to Japanese controls, Sri Lankan participants reported remarkably low urinary cadmium levels, and no significant correlation was observed between the studied biomarkers and cadmium urine excretion. Familial clustering analysis with patients and their relatives strongly suggested a potential genetic predisposition to this disease [47].

In summary, the use of novel biomarkers in identifying CKDu in Sri Lanka is very promising. Several biomarkers have been utilized and a few of them (e.g., KIM-1, NGAL, A1M) can be used in a broad clinical set up. However, the sensitivity and specificity of the biomarkers warrant further studies. Additionally, most studies were focused on CKDu endemic locations, and the levels of biomarkers in healthy adult populations and children among the rural farming communities remain unclear. The use of novel markers in Sri Lanka may largely depend on future longitudinal cohort studies, where the markers’ prognostic value could be further evaluated.

### 5.3. India

Characteristics of CKDu India share some similarities with that of CKDu patients in Sri Lanka and Mesoamerica. For example, a recent study with 2210 participants above 18 years of age reported a high prevalence of 18.23% of CKD in rural Indian communities in the Uddanam region. Importantly, 73% of the diagnosed CKD patients did not have diabetes, long-standing hypertension, or significant proteinuria (>1), indicating a higher prevalence of CKDu [26]. Most were farmers, and 55.7% of them were women. A creatinine level above 1.2 mg/dL was seen in 13%, and 59% were males. Low eGFR (<60) was seen in 13.9% of the population. Urinary protein creatinine ratio (more than 0.2) was seen in 9.04% and when eGFR and proteinuria were both taken together, 18.2% of the population was deemed to have CKD. However, none of India’s published clinical studies on CKDu appeared to have utilized any of the novel biomarkers described in the current review.

### 5.4. Egypt

CKD has been identified in Egyptian communities via several studies. In a cohort of 587 children, CKD patients with stage one to five were detected. Obstructive uropathy, primary glomerulonephritis, reflux/urinary tract infection, aplasia/ hypoplasia, and familial/metabolic diseases were associated with renal dysfunction, while 20.6% of participants had no identified etiology, classifying them in CKDu [87]. In another study with 1004 known CKD patients in the El-Sharkia Governorate in Egypt, hypertension and diabetes were the main causes found, while 17.7% of participants were classified as CKDu [88]. However, no novel biomarker-based studies on CKDu in Egyptian communities are available in the literature.

### 5.5. Indonesia

A recent cross-sectional study with male rice farmers in Karawang and Bogor Regency, in West Java Province in Indonesia, evidenced the presence of CKDu. Out of the 354 healthy male farmers, the total CKD prevalence was 24.9%, and CKDu prevalence was 18.6% [11]. The determination was based on conventional markers, and no novel biomarker-based studies were reported from Indonesia.

## 6. Conclusions

CKDu has been clinically characterized using both novel and conventional biomarkers in many of the impacted regions. Within the spectrum of clinical findings, a variety of factors, including heavy metals, heat stress, agrochemicals, nephrotoxins, infections, geography, and socio-economic factors appear to be contributing to CKDu. The significance of each factor may vary depending on the patient population, and the continuous use of conventional and novel biomarkers will contribute to an improved understanding of this disease.

To date, SCr, eGFR, Cys-C, and ACR have been used extensively for screening. Considering studies on different communities with CKDu, a decline in eGFR was observed along with elevated SCr and BUN levels. Although these markers appear valid when distinguishing prospective patients, they are suboptimal when evaluating kidney health in cross-sectional studies. To this end, longitudinal studies will identify and validate biomarkers that may predict renal outcomes. For example, in some Mesoamerican studies, a decline in renal function is characterized by reduced eGFR, increased SCr, BUN, and novel biomarkers. However, these studies did not examine chronicity or progressive decline in renal function over a considerable period. It is possible that participants of some of the Mesoamerican studies could have had reduced renal function with normal serum creatinine at the entry to the study, and the elevation of serum creatinine in response to dehydration may have been an epiphenomenon of underlying CKD rather than the cause of CKDu. Therefore, more longitudinal studies with longer follow-up using multiple renal biomarkers will help unravel such confounding factors and provide more insights into the validity of the marker and potential causative factors.

In addition to early predictive markers, it is critical to have markers with higher sensitivity and specificity to define CKDu pathology. The most common traditional markers such as SCr, proteinuria, ACR, and eGFR are quite useful in determining renal impairment and renal function variation over time. However, these are generic markers that indicate renal impairment and are not informative on the damage’s nature. The novel biomarkers discussed in this review have begun to gain importance in the clinical setting for their rapid and site-specific expression. For example, biomarkers such as KIM-1 and NGAL become detectable in renal damage earlier than detectable changes in creatinine and eGFR. NGAL is especially becoming recognized as a biomarker for monitoring CKD progression. However, each biomarker has different pathologies, and therefore a panel of defined biomarkers would be more revealing in clinical screening for CKD or CKDu. Validation and establishment of reference levels for novel biomarkers such as CysC, NGAL, and KIM-1, some of which are potentially important in the diagnosis of prerenal dysfunction [89], and other lesser-known biomarkers such as insulin-like growth factor binding protein (IGFBP) that could possibly predict renal recovery [90], would be useful in demonstrating the AKI-CKD continuum in agricultural workers.

While it is clear that the use of strategic batteries of biomarkers with diagnostic, monitoring, predictive, prognostic and/or susceptibility/risk characteristics is needed to detect better, monitor, and risk screening CKDu endemic regions, costs of these may become prohibitive. Many of the novel biomarkers discussed here remain inaccessible in low resource settings (Supplementary Materials Table S1), primarily due to lack of equipment and high costs per sample. In fact, to date, no novel biomarker has been incorporated beyond the research level into clinical laboratories for public access at affordable rates. The currently available analytical techniques, most commonly Enzyme Linked Immunosorbent Assays (ELISA), are expensive and time-consuming compared to conventional markers. Therefore, although a large spectrum of potential novel biomarkers has emerged in recent years, their value in clinical application is yet to be established, especially in the context of CKDu. This indicates that, in addition to advancements in high throughput technologies (e.g., proteomics), sample conditioning, and analytical trajectories in search of superior biomarkers for renal diseases, efforts to reduce the cost of analysis of existing novel biomarkers are also critical. For example, with the emergence of CKDu cases in Sri Lanka, governmental and non-governmental efforts helped implement clinical laboratory facilities in rural settings to assess conventional markers such as creatinine and microalbumin. Upon recognizing the potential value of novel biomarkers, local authorities and practitioners can play an important role in soliciting equipment and resources to be added to these locations enabling other biomarker assays.

Finally, as CKDu is largely non-proteinuric with normal creatinine levels in the earlier phases, conventional tests are likely missing opportunities to diagnose this disease early. Although CKDu is commonly diagnosed in adults, children in CKDu prevalent communities are also at risk, as shown in Nicaragua [71,72] and Sri Lanka [74]. This critical situation highlights the paramount importance of detailed and intensive studies for characterizing the early development of CKDu. Longitudinal studies involving children cohorts may provide ideal follow-up studies to identify biomarkers with improved predictability and may complement ongoing efforts to validate the prognostic power of novel renal biomarkers before translation into broad clinical practices. Nonetheless, we emphasize that the use of these markers in CKDu impacted communities warrants greater attention, since they may be the key to mitigating this complex disease.

## Figures and Tables

**Table 1:** Examples of novel kidney injury biomarkers used in CKDu around the globe with their characteristics and clinical significance. (Abbreviations: KIM-1—kidney injury molecule -1; NGAL—neutrophil gelatinase-associated lipocalin; A1M—α1-microglobulin; B2M—macroglobulin; NAG—N-acetyl-beta-D-glucosaminidase; CLU—clusterin; OPN—osteopontin; CysC—cystatin-C; IL-18—Interleukin 18; MCP-1—monocyte chemoattractant protein).

Bio- Marker	Biochemical Significance	Site of Release in the Kidney	Clinical Significance
CLU	Referred to as apolipoprotein J (Apo J). 75–80 kDa glycoprotein.	Proximal tubule Dedifferentiated tubular cells Distal tubule	Elevated urinary expression observed in rat models of tubular proteinuria, but not in glomerular proteinuria.
CysC	Low molecular weight protein (13 kDa). Freely filtered at the glomerulus, subjected to reabsorption and catabolism, but not to tubular secretion.	Glomerulus Proximal tubule	Sensitive serum marker of GFR Prognostic marker of adverse outcomes such as renal replacement therapy. A predictive biomarker of cardiovascular events in patients with kidney diseases.
KIM-1	A Highly conserved phosphatidylserine receptor of 38.7 kDa with a mucin-rich extracellular region.	Proximal tubule Dedifferentiated tubular cells	Proximal tubular injury marker A sensitive and early diagnostic marker of renal injury. Associated with tubule-interstitial fibrosis, tubular damage, and inflammation.
NAG	A proximal tubule lysosomal enzyme.	Proximal tubule	A sensitive and robust indicator of AKI Increased in impaired glucose tolerance, rheumatoid arthritis, and hyperthyroidism.
NGAL	25 kDa lipocalin superfamily protein. A glycoprotein bound to matrix MMP-9 in human neutrophils.	Proximal tubule Distal tubule	A sensitive and early diagnostic marker of renal injury and AKI. Marked upregulation in proximal tubules within 3 h of ischemia/reperfusion injury. Also expressed in salivary glands, uterus, prostate, trachea, lung, stomach in addition to the kidney.
OPN	44 kDa extracellular matrix glycol-phosphoprotein. Produced in high levels in bone tissue and epithelial cells.	Proximal tubule Loop of Henle Distal tubule	Marker of nephrotoxicity. Expressed with inflammation and tubulo-interstitial fibrosis in progressive idiopathic membranous nephropathy, crescentic glomerulonephritis, IgA nephritis, and diffuse proliferative lupus nephritis. Involved in remodeling and calcification of bones, cell adhesion, migration, and survival, immune responses and tumorigenesis.
A1M	27 -30 kDa glycoprotein primarily synthesized by the liver. Detectable in human urine, serum and cerebrospinal fluid. Freely filtered at the glomerulus, subjected to complete reabsorption and catabolized by proximal tubular cells.	Proximal tubule	Elevated levels in urine reflects proximal tubular injury or dysfunction. Urinary levels increase with age. Plasma level varies with age, gender, liver diseases, ulcerative colitis, HIV infection, and mood disorders.
B2M	13.7 kDa light chain MHC-I protein Expressed on all nucleated cells, serum, urine, and synovial fluid. Filtered at the glomerulus and almost entirely reabsorbed and catabolized by the proximal tubular cells.	Proximal tubule	Tubular injury marker in AKI and CKD. Increase in serum reflects the decrease in glomerular function in CKD. Elevated in cadmium toxicity, following cardiac surgery, liver and renal transplantation and idiopathic membranous nephropathy.
IL-18	18 kDa proinflammatory cytokine, activated via cleavage by caspase-1. Induced and cleaved mainly in the proximal tubules in human kidney and released into the urine.	Proximal tubule	Elevated urinary IL-18 levels are apparent in patients with kidney injury.
MCP-1	8.7 kDa protein of Chemotactic cytokine (chemokine) family. Produced in renal cells in response to proinflammatory cytokines.	Glomerular mesangial cells Podocytes Tubular epithelial cells	Expressed by renal cells following renal injury. Urinary MCP-1 is upregulated in inflammatory renal disease and diabetic nephropathy.

## Supplementary Materials

The following are available online at https://www.mdpi.com/1660-4601/17/24/9522/s1, Table S1: Current utilization, cost implications, and practical considerations in biomarker utilization in CKDu impacted populations.

## Abbreviations

A1M	α1-microglobulin.
ACR	albumin–creatinine ratio
AFB1	aflatoxin-B1
AKI	acute kidney injury
B2M	β2- macroglobulin
BUN	blood urea nitrogen
CKD	chronic kidney disease
CKDu	chronic kidney disease of unknown etiology
CLU	clusterin
CysC	cystatin-C
DALYS	disability-adjusted life years
eGFR	estimated glomerular filtration rate
FDA	food and drug administration
FN1	fibronectin- 1
GCLC	glutamate-cysteine ligase catalytic
GFR	glomerular filtration rate
GSTM1	glutathione-S-transferase mu 1
HGF	hepatocyte growth factor
HIV	human immune virus
IGFBP	insulin-like growth factor binding protein
IL-18	interleukin-18
KDIGO	Kidney disease: improving global outcomes
KIM-1	kidney injury molecule -1
L-FABP	liver type fatty acid binding protein
KLK1	kallikrein-1
NAG	N-acetyl-beta-D-glucosaminidase
NGAL	neutrophil gelatinase-associated lipocalin
NIH	National Institutes of Health
NLR	nod-like receptor
NLRP3	NLR family pyrin domain containing 3
OPN	osteopontin
Pi-GST	glutathione S-transferase
RBP	retinol binding protein
RRT	renal replacement therapy
SCR	serum creatinine
TIMP-2	tissue inhibitor metalloproteinase-2
uNGAL	urinary neutrophil gelatinase-associated lipocalin
μUNPD	micro-urine nano particle detection
