# Supplementary material for: The Utility of Novel Renal Biomarkers in Assessment of Chronic Kidney Disease of Unknown Etiology (CKDu): A Review
Source: Int J Environ Res Public Health. 2020 Dec 18;17(24):9522. doi: 10.3390/ijerph17249522 (PMC7766480; doi:10.3390/ijerph17249522)
Supplement: Supplementary file 1 [file ijerph-17-09522-s001.pdf]

Table S1. Current utilization, cost implications, and practical considerations in biomarker utilization in CKDu impacted populations.

**Biomarker and locations of current utilization**	**Method of determination**	**Minimum cost per sample for duplicate measurements**	**Advantages**	**Drawbacks**
Serum creatinine	Colorimetric methods using clinical chemistry analyzer	<$1.00	- Rapid test - takes less time (5-10 minutes)<br>- Tests are easily accessible in a rural setting<br>- Typically, available in clinical laboratories in rural hospitals	- Merely indicates renal abnormality without specific indication on site of injury<br>- Not sensitive enough to characterize early renal damage
	ELISA (commercial kits)	~$6	- High accuracy in determination	- Time consuming (4-5 hours)<br>- Commercial kits are developed for *In vitro* Diagnostic (IVD) purposes<br>- Not available in rural clinical laboratories and reduced accessibility for general public.
Urinary microalbumin	Turbidimetric methods using clinical chemistry analyzer	~$2	- Takes less time (5-10 minutes)<br>- Tests are easily accessible to public<br>- Available in clinical laboratories	- Merely indicates renal abnormality without specific indication on site of injury<br>- Not sensitive enough to characterize early renal damage
	ELISA (commercial kits)	~$10	- High accuracy in determination	- Time consuming (4-5 hours)<br>- Commercial kits are developed for IVD purposes<br>- Not available in clinical laboratories and not accessible for general public.
Cystatin C	Colorimetric methods using clinical chemistry analyzer	Undetermined		
	ELISA (commercial kits)	~$15	- High accuracy in determination	- Time consuming (4-5 hours)

				- Commercial kits are developed for IVD purposes<br>- Not available in most clinical laboratories
KIM-1	ELISA (commercial kits)	~$8	- High accuracy in determination<br>- Indicates early renal damage<br>- Give specific indication on site of renal injury	- Time consuming (4-5 hours)<br>- Commercial kits are developed for IVD purposes<br>- Not available in most clinical laboratories
NGAL	ELISA (commercial kits)	~$9		
Cystatin C	ELISA (commercial kits)	~$9		
